# Use of Finite Elements Analysis for a Weigh-in-Motion Sensor Design

**DOI:** 10.3390/s120606978

**Published:** 2012-05-25

**Authors:** Rigobert Opitz, Viorel Goanta, Petru Carlescu, Paul-Doru Barsanescu, Nicolae Taranu, Oana Banu

**Affiliations:** 1 ROC Systemtechnik GmbH, Elisabethstrasse 69, A-8010 Graz, Austria; E-Mail: rigobert.opitz@rocgmbh.com; 2 Mechanical Faculty, Technical University “Gheorghe Asachi” of Iasi, Strength Materials Department, Bd. D. Mangeron, nr. 61-63, IaŞi 700050, Romania; E-Mail: paulbarsanescu@yahoo.com; 3 Faculty of Agriculture, Department of Agricultural Technology, “Ion Ionescu de la Brad” University of Agricultural Sciences and Veterinary Medicine IaŞi, 3 Mihail Sadoveanu Alley, IaŞi 700490, Romania; E-Mail: pcarlescu@uaiasi.ro; 4 Department of Civil and Industrial Engineering, Faculty of Civil Engineering and Building Services, Technical University “Gheorghe Asachi”, Iasi, 43 D. Mangeron Blvd., Iasi 700050, Romania; E-Mail: taranu@ce.tuiasi.ro; 5 Department of Structural Mechanics, Faculty of Civil Engineering and Building Services, Technical University “Gheorghe Asachi”, Iasi, 43, D. Mangeron Blvd., Iasi 700050, Romania; E-Mail: oana_banu@ce.tuiasi.ro

**Keywords:** weigh in motion, WIM sensor, shear force sensors, optimization, sensitivity, durability

## Abstract

High speed weigh-in-motion (WIM) sensors are utilized as components of complex traffic monitoring and measurement systems. They should be able to determine the weights on wheels, axles and vehicle gross weights, and to help the classification of vehicles (depending on the number of axles). WIM sensors must meet the following main requirements: good accuracy, high endurance, low price and easy installation in the road structure. It is not advisable to use cheap materials in constructing these devices for lower prices, since the sensors are normally working in harsh environmental conditions such as temperatures between −40 °C and +70 °C, dust, temporary water immersion, shocks and vibrations. Consequently, less expensive manufacturing technologies are recommended. Because the installation cost in the road structure is high and proportional to the WIM sensor cross section (especially with its thickness), the device needs to be made as flat as possible. The WIM sensor model presented and analyzed in this paper uses a spring element equipped with strain gages. Using Finite Element Analysis (FEA), the authors have attempted to obtain a more sensitive, reliable, lower profile and overall cheaper elastic element for a new WIM sensor.

## 1. Modelling and Optimization by Using Finite Element Analysis (FEA)

Modern design methods require, in certain phases, the use of FEA that enables the achievement of safe projects in terms of reliability and durability. There are certain advantages when using the FEA in the design process [1]:

- Decreased design costs;
- Reduction of manufacturing costs;
- Material savings;
- Weaknesses identification;
- Improvement of the project quality;
- Components and assembly optimization.

Several steps are necessary to optimize the components' shape and size by using FEA. In the first stages, when quick results are required, a certain degree of error is accepted. In the next stages, very accurate results are needed, even if it demands more running time. During these final stages, a more refined mesh is necessary. Usually, the management of FEA from the design stage for the determination of the final shape and dimensions is based on the designer intuition and judgement.

**Figure 1.**
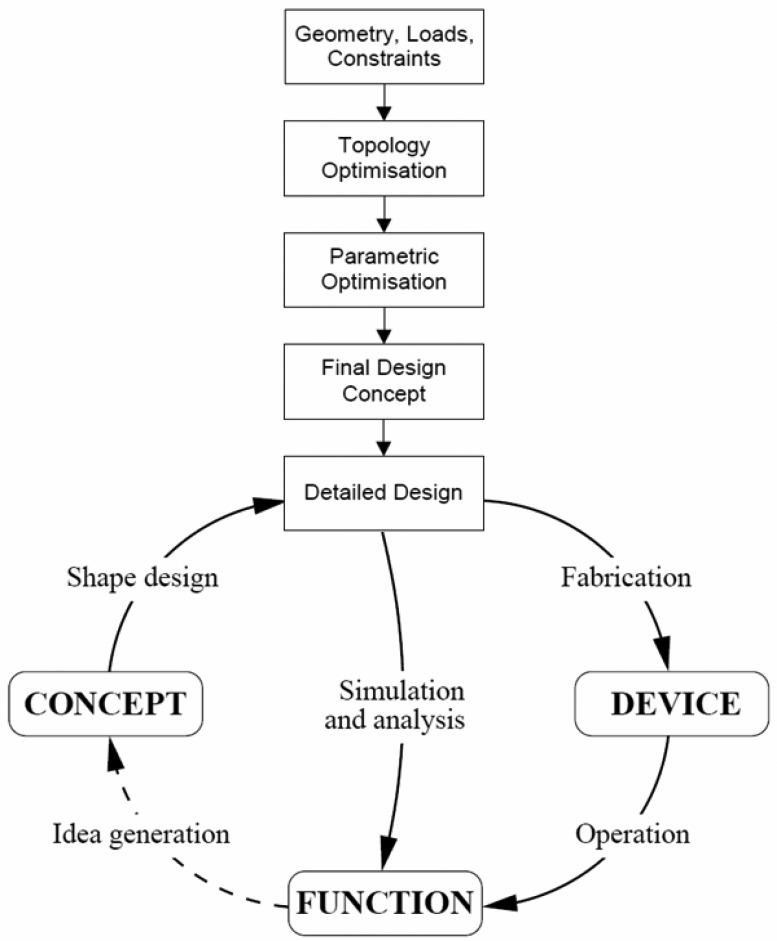
The product development iteration cycle [2].

A flow chart containing the iterative steps for product development is presented in Figure 1 [2] where the necessary steps for the determination of the components' final shape and dimensions by using FEA are illustrated. Although the Finite Element Method (FEM) implies high computing power and usually ensures a reasonable precise analysis of complex structures, the provided solutions can be sometimes affected by certain distortions, produced by various causes [3].

The disadvantage of using the FEM for constructive shape optimization is that it is not clearly revealed how strains and stresses are influenced by important variables such as material properties, geometric characteristics, fixing solutions, *etc.* [4]. Even the analyst may introduce some errors.

Perhaps the most important function in the modelling and optimization process is the experience and intuition of the designer in using the FEA software, his/her ability to establish a good strategy from the beginning, using also some experimental results to validate the numerical data obtained from the FEA [5].

## 2. Weigh-in-Motion Sensors

Weigh-in-motion (WIM) sensors are currently utilized for weight on wheel, on axle and gross vehicle weight measurement, as well as for traffic monitoring. These sensors can detect the overloaded axles of vehicles that have a major contribution to pavement damage [6]. They may also contribute to establishing an efficient and fair transport system, protecting the road infrastructure.

There are three main WIM sensors on the market nowadays: two are based on electrical gages (single load cell and bending plate) and one on piezoelectric technique. Studies made by several authors have shown that WIM sensors with electrical strain gages give the best accuracy (±6% and ±10% respectively) and the longest life, but they are also the most expensive. Piezo WIM sensors have a small cross section and are cheaper. Sensors' cost and their installation cost in the road are proportional to their cross section. Especially the sensor thickness affects the installation costs in the road. The sensor presented in this paper combines the advantages of both categories of WIM sensors on the market because it uses electrical strain gages and has a small cross section (similar to piezo WIM sensors). The future development of the WIM sensors will imply the following practical applications:

- Improvement of the dynamic weighing accuracy at high sample rates;
- Improvement of the determination of the European Standard Axle Load (ESAL) factor which will contribute to better pavement design, protection and cost reduction;
- Improvement of vehicle detection and classification;
- Automated overload detection and enforcement;
- Decreasing of the sensor's cross section, especially its thickness, since the installation costs are proportional to the slot dimensions in the road structure (especially its depth);
- The new generation of WIM systems will also be able to measure the footprint dimensions and to detect flat tires.

The design and optimization of the spring element (using FEA) of a new WIM sensor, proposed by ROC Systemtechnik GmbH is presented in the following sections [6–8].

## 3. Initial WIM Sensor Model (7.1 mm Thickness of the Elastic Element)

An innovative WIM sensor proposed by the ROC Company has been conceived and it shows the following advantages [9]:

- Small cross section and flat design (90 mm ×24 mm);
- Good accuracy and repeatability;
- Good reliability;
- Moderate price;
- Low installation cost.

**Figure 2.**
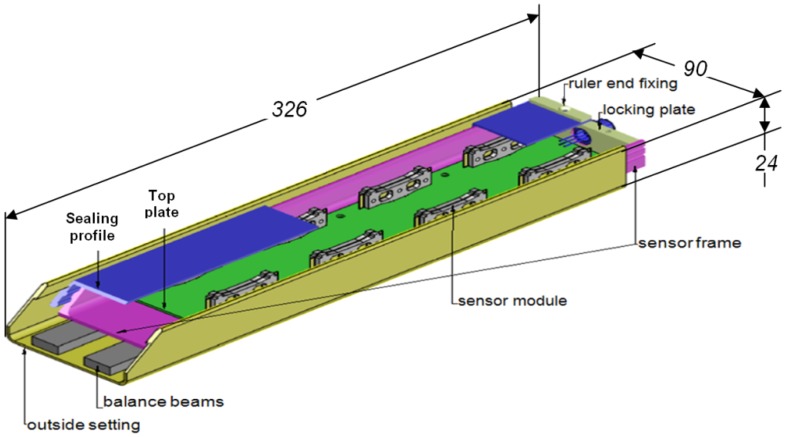
A WIM sensor box.

Each sensor box designed as a matrix of 16 measurement points contains eight sensor modules representing the shear beam spring elements (Figure 2). In a WIM system, each lane of the road is equipped with a line of sensors, consisted of several WIM sensor boxes covering the whole lane width.

**Figure 3.**
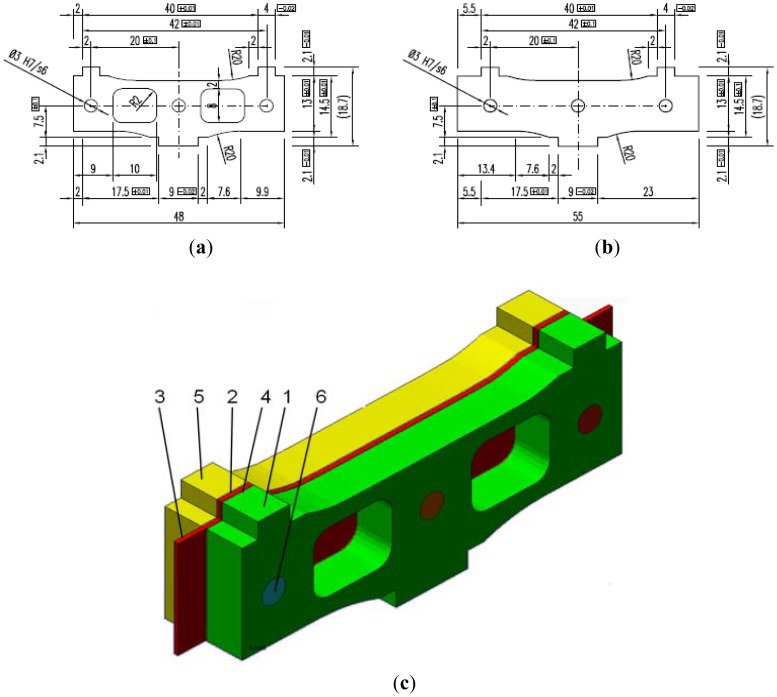
Components and assembly of a sensor module: (**a**) Outer plates; (**b**) Central elastic plate; (**c**) Assembly of sensor module [10].

The initial design of a sensor module is presented in Figure 3. The thickness of the module components and their elastic characteristics are given in Table 1. The overall dimensions of this assembly are 48 mm × 18.7 mm × 7.1 mm. It is composed of two outer plates, (1) and (5) as shown in Figure 3(a), and a central elastic plate (3), Figure 3(b), bonded together by the adhesive layers (2) and (4), Figure 3(c). To avoid the relative displacements between the three plates, three pins (6) are also inserted in the assemblage. The bottom part of the sensor module is embedded in the sensor box and the upper part is loaded by the tires running on the top pad of the box. The central elastic plate (3) is equipped with strain gages and their outputs are sent to a data acquisition system.

## 4. Modelling and Data Processing in the FEA Stage

### 4.1. Model and Materials

**Figure 4.**
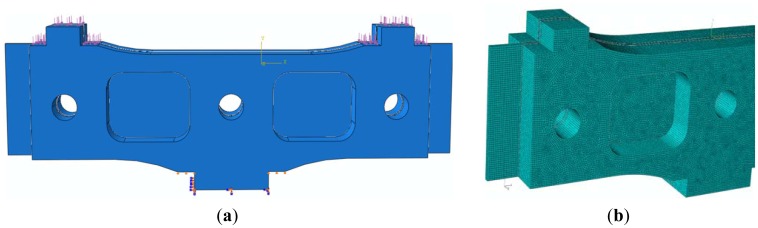
Loading, boundary conditions and meshing of the elastic element: (**a**) Loading and boundary conditions; (**b**) Meshing of the elastic element (detail).

Finite Element Analysis in the elastic range, under static loading conditions, has been performed using the ABAQUS v6.8 software. The elastic element has been considered to be embedded at the bottom part and leaned on the flat surfaces near the lower leg. The maximum load of 6,000 N, corresponding to the heaviest vehicle, was equally divided on the two arms of the elastic element and uniformly distributed on the top surfaces and the flat surfaces in their vicinity (Figure 4). The three sensor plates are fixed with adhesive and three pins. The boundary conditions on the pin holes surface were connection (limited to longitudinal displacement in the direction of OZ axis and rotation of OX, and OY axis). Initially, the outer plates and the central elastic plate have been made of stainless steel 1.4548.6 (X5CrNiCuNb17-4-4) Bohler N700, having the yield point σ_0.2_ = 1,170 MPa, considered a homogeneous and isotropic material.

The meshing has been performed by using a hybrid network, with finite elements (FE) predominantly “brick” type (Figure 4(b)), resulting a number of 172,202 finite elements. The elements distribution was considered uniform throughout the model, with the same increasing factor [11]. The above mentioned number of finite elements has been obtained after performing three consecutive trials with an increased number of FE (73,181; 112,300; 172,202), so that the meshing error was eliminated and the running time acceptable [12].

Two loading cases have been considered:

*Case 1*: symmetric loading of 3,000 N on both arms of the sensor module (Figure 4(a));
*Case 2*: asymmetric loading, *i.e.*, only one arm of the sensor is loaded with a force of 3,000 N and the displacement of the other arm on OY direction is restrained. This could be the case when the edge of the tire acts on the arms of two different neighbouring sensors.

### 4.2. Symmetric Loading

**Figure 5.**
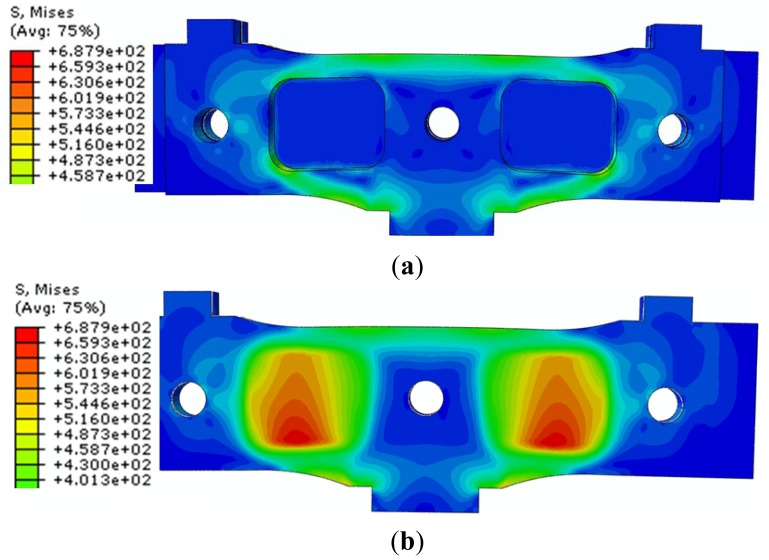
State of von Mises stresses for symmetric loading (σ_max_ = 687.92 MPa): (**a**) Assembled sensor module; (**b**) Central elastic plate.

The distribution of “*von Mises stresses*” is presented below:

- stresses in the assembled sensor module (Figure 5(a));
- stresses in the central elastic plate (Figure 5(b)).

Note that the strain gages have been located in the region with anticipated maximum stresses, namely on the central elastic plate area (σ_max_ = 687.92 MPa). The main direction of the maximum normal stresses in the strain gages area is at 45°, and the strain gages are oriented with the filaments in this direction.

### 4.3. Asymmetric Loading

**Figure 6.**
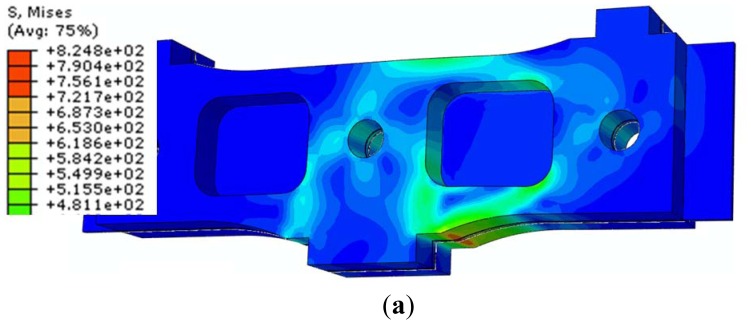

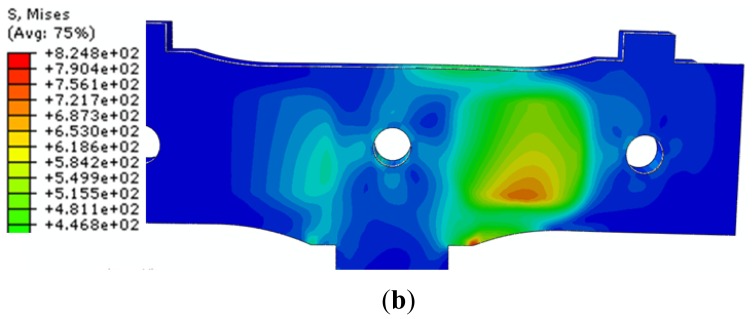
The state of von Mises stresses for asymmetric loading case (σ_max_ = 824.787 MPa): (**a**) Sensor module; (**b**) Central elastic plate.

In the second loading case, only one force equal to 3,000 N is applied on one of the sensor module arms. In Figure 6 the state of von Mises stresses on the sensor module and on central elastic plate respectively, is illustrated. In this situation, the maximum von Mises stress is σ_max_ = 824.787 MPa.

This stress is localized on the central elastic plate, in the strain gages area. There is a significant increase of stresses, from 687.92 MPa, for symmetric loading case, to 824.787 MPa, for asymmetric loading case. As it will be shown below, this latest value of stresses is larger than the fatigue strength of the 1.4548.6 (X5CrNiCuNb17-4-4) Bohler N700 steel type.

## 5. Sensor Fatigue Analysis

In Table 2, the main normal stresses and maximum displacements for symmetric and asymmetric loading cases are summarized. Note that the most severe condition is the asymmetric loading case.

FEA has been carried out for static loading conditions and the fatigue analysis is presented in the following. The ratio between the minimum and maximum stress, with the sign taken into account, is known as *cycle characteristic* or the *coefficient of cycle asymmetry*, denoted by *R*. To estimate the fatigue strength of steel, an empirical relationship has been used, appropriate for the steel types with an ultimate tensile stress between 1,200 MPa and 1,800 MPa [13]:

(1) $$\sigma_{-1}=400+\frac{\sigma_{u}}{6}$$

where:

- σ_−1_ is the fatigue limit for symmetric cycle (R = −1);
- σ_u_ is the ultimate tensile stress.

The symmetric cycle (R = −1) is the most dangerous scenario for materials fatigue. Accordingly, if a component is designed to resist a long lasting load with a symmetric cycle, it will be even more able to resist for any cycle with the same amplitude and different coefficient of cycle asymmetry *R*. The elastic element of the WIM sensor is loaded with a fluctuating cycle (zero base or R = 0). Usually, the fatigue strength for fluctuating cycle σ_0_ is greater than for symmetric cycle σ_−1_. Constant cycle amplitude has also been assumed as if all vehicles would have been trucks with all axles loaded to maximum.

Using Equation (1) for the stainless steel 1.4548.6 (X5CrNiCuNb17-4-4) Bohler N700, which has the ultimate tensile stress σ_u_ = 1,310 MPa, a fatigue strength σ_−1_ = 618.33 MPa for symmetric cycle is obtained.

Utilizing the data presented in Table 2, it can be seen that the absolute minimum value of normal stress σ_min_ (711.383 MPa) is greater than the fatigue strength for a symmetric cycle (618.33 MPa):

(2) $$\left|\sigma_{\min}\right|>\sigma_{-1}$$

Equation (2) signifies that the sensor may not be able to resist to an infinite number of cyclic stresses. Therefore, a redesign of the initial version of the sensor has been recommended. The main factors affecting the fatigue life are: the material volume, the roughness and the stress concentrators. The sensor volume is less than that of a sample for fatigue test, characteristic that has a favorable influence on the sensor endurance. In addition, the critical surfaces of the piece have a small roughness (fine grinding). FEA has been focused on decreasing the stress concentrations. Therefore the stress concentration factors have only very moderate values in the critical areas. Consequently, the global influence of the above three mentioned factors it is also small. The improvement of the WIM sensor fatigue behavior can be achieved by:

- Changing the shape, to reduce the local maximum stresses;
- Choosing a steel type with a larger fatigue strength;
- Creating surfaces with lower roughness;
- Adoption of all above solutions.

At the same time, the strain in the strain gages area has to be kept large enough to have a good sensitivity.

## 6. The New Design of the WIM Shear Beam Sensor

**Figure 7.**
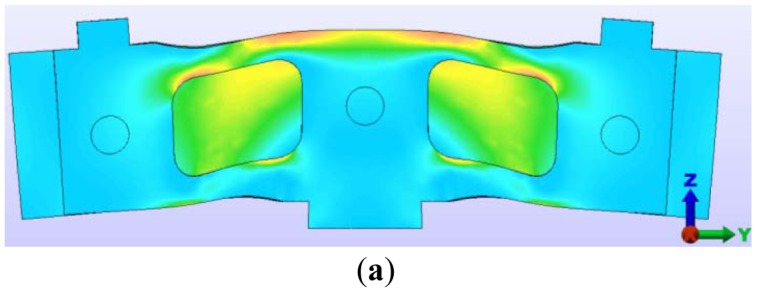

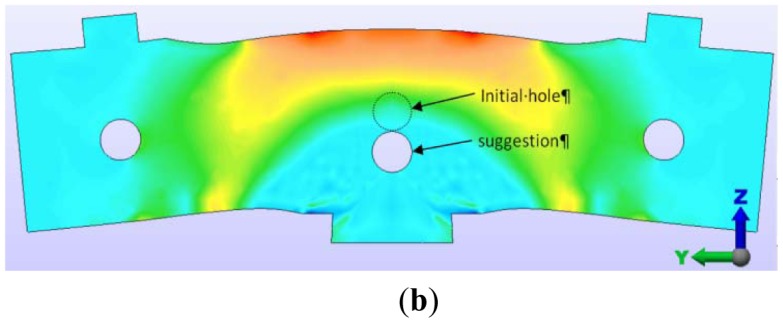
State of main stresses in the deformed sensor module and central elastic plate.

Using the symmetric loading case and the boundary conditions presented above, the state of the main stresses in the deformed sensor module, and central elastic plate has been obtained and it is illustrated in Figure 7. The central elastic plate and the two outer plates are bonded together by the two adhesive layers and three pins. For this reason, between these three plates there are no relative displacements.

**Figure 8.**
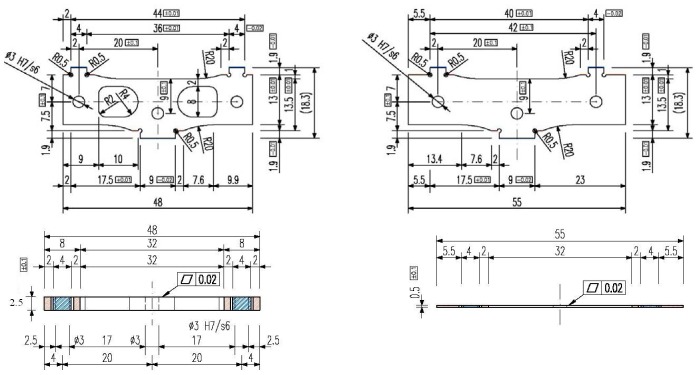
The new WIM shear beam sensor design.

Note that the central pin hole is located in an area with important stress values. Considering the maximum stress values obtained from the FEA, the following modifications have been adopted, to increase the sensor endurance:

- The central hole has been relocated by moving it below with a distance of one diameter, in an area with lower stresses, (Figure 7(b));
- Higher connection radius of the two clearances in the outer plates (to the central axis), as one can see in Figure 8(a);
- The leaning surfaces must be machined by grinding and for this reason special releases were provided at the inner corners;
- The top surface, where there are high stresses, must be also machined by grinding.

In addition to the above recommendations, the stainless steel Sandvic 7C27M02, with superior characteristics (σ_u_ = 1,600 MPa, a good fatigue strength *etc.*), has been selected for the elastic element of the sensor module.

The following dimension changes have been proposed since this new steel type is produced only in strips, in a small range of sizes:

- outer plates thickness will be of 2.5 mm instead of 3.18 mm;
- accordingly, the thickness of the elastic element assembly becomes 5.74 mm (the thickness of the central elastic plate and adhesive layers remains the same).

The reduction of the plate section will also determine a larger output of the strain gages, *i.e.*, a sensitivity increase. The new WIM shear beam sensor design is shown in Figure 8. Considering the above changes, a new FEA of the elastic element is necessary.

## 7. Analysis of the Final Version of WIM Sensor

### 7.1. Finite Element Analysis

The new FEA will take into account the following:

- Only the asymmetric loading case has been considered, because it generates higher stresses, as it was established in the first analysis;
- The deformed central elastic plate should not touch the bottom surface of the sensors box.

**Figure 9.**
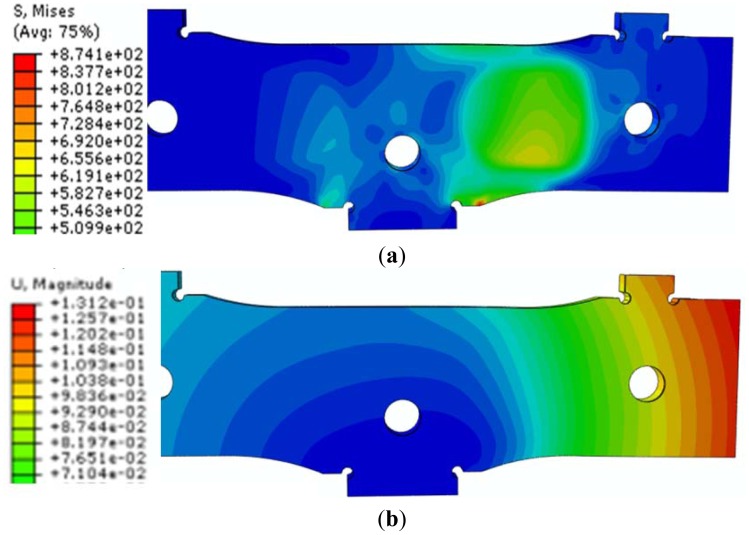
State of von Mises stresses and displacements in the central elastic plate: (**a**) von Mises stresses (σ_max_ = 874 MPa); (**b**) Displacements (**δ**_max_ = 0.131 mm).

The same loading and boundary conditions have been used for the new FEA. As a result of this FEA, new data on the state of “*von Mises stresses*”, main stresses (σ_11_ and σ_22_), strains, and displacements have been obtained (Figure 9).

In Table 3, the maximum and the minimum stress, as well as the maximum displacement (located at the free ends of the central elastic plate) for the new design of WIM sensor and asymmetric loading case are given.

### 7.2. Fatigue Computation of the New WIM Sensor

Using Equation (1) for the Sandvic 7C27M02 stainless steel (σ_u_ = 1,600 MPa), the fatigue strength for symmetric cycle is obtained as σ_−1_ = 667 MPa. Comparing this value with the absolute value of the maximum stress from Table 3, one may notice that for the new design, the absolute minimum value of normal stress σ_min_ (658.179 MPa) is lower than the fatigue strength for a symmetric cycle:

(3) $$\left|\sigma_{\min}\right|=658\text{MPa}<\sigma_{-1}=667\;\text{MPa}$$

Due to the inequality (3), it can be stated that the new WIM shear beam sensor has an unlimited fatigue life. From Table 2 and Table 3 it can be seen that the maximum displacement is slightly larger in the case of new design (0.131 mm compared to 0.121 mm).

**Figure 10.**
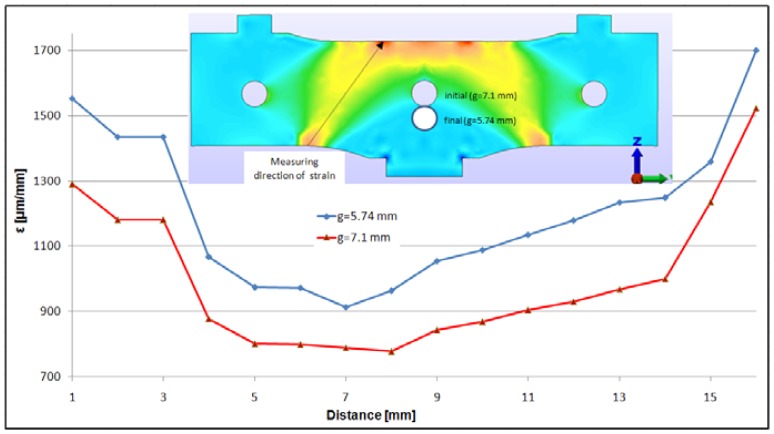
Central elastic plate—Strain variation on the measurement direction (strain gage direction) for the new WIM sensor design.

In Figure 10, the strains on the strain gage direction are presented. Comparing the two solutions it can be seen that, for the new design, the strains are higher at any point on the strain gage grid. This causes a higher output of the strain gages and, of course, a better sensitivity.

A complete sensor box includes eight elastic elements mounted in two rows. The nominal force on an elastic element is 3 kN and the sensitivity is 1 N. The nominal load on a box sensor is 24 kN. The cross section of a box sensor is 90 × 25 mm.

**Figure 11.**
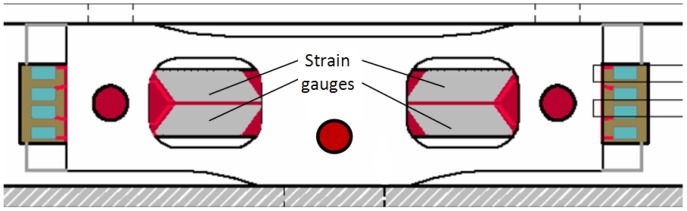
Strain gauge transducer positioning.

Strain gauges were chosen so as to fit in rectangular area made in the outer plates, Figure 11. On the other hand, one of the strain gauges directions coincides with the maximum stress direction, shown in Figure 10. In these conditions, the sensitivity to measuring with strain gauges, increases considerably.

**Figure 12.**
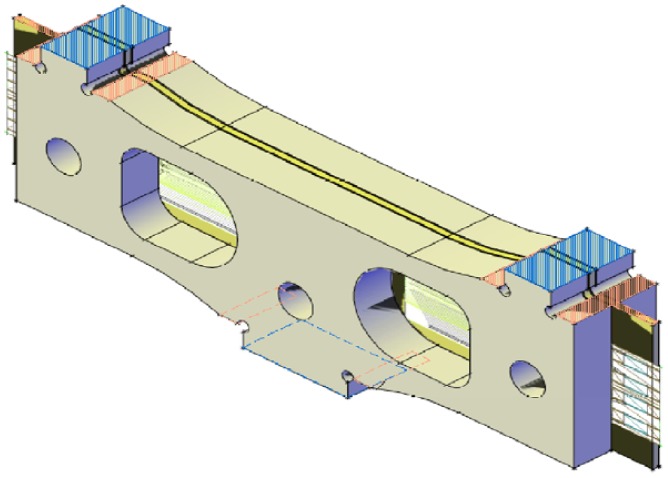
Final version of the WIM sensor module.

**Figure 13.**
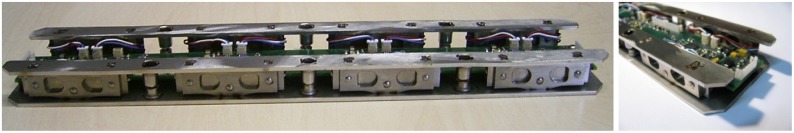
Assembled WIM sensor module [14].

A 3D drawing of the final version of the WIM sensor module is shown in Figure 12. The WIM sensor has been finally built using the new design (Figure 13).

## 8. WIM Sensor *In-Situ* Measuremets

The designed sensors (Figure 12) have been mounted eight in two rows in a sensor box (Figures 2 and 12), together with the electronics. The sensor boxes are mounted in series, making up lines of sensors crossing the road. The assemblage has a data acquisition system, data processing software and a wireless data transmission. A line of sensors has been mounted into the road structure in Graz (Austria) and on the highway near Rosenheim (Germany). In Table 4, an example of data supplied by the WIM system is presented. The sensor measure and records the weight on each wheel and on each axle of the vehicle at the highway travelling speed. The date, time and the data provided by the WIM sensor (the number of axles, the weight on each axle, the total gross weight, the distance between axles, the overload on each axle) are displayed, as well as data provided by other sensors (the speed). Using adequate software, the vehicle category which passed over the sensor can be identified (column “Type” from Table 4). With the help of video cameras, the licence plate number of each vehicle can be recorded and identified.

**Figure 14.**
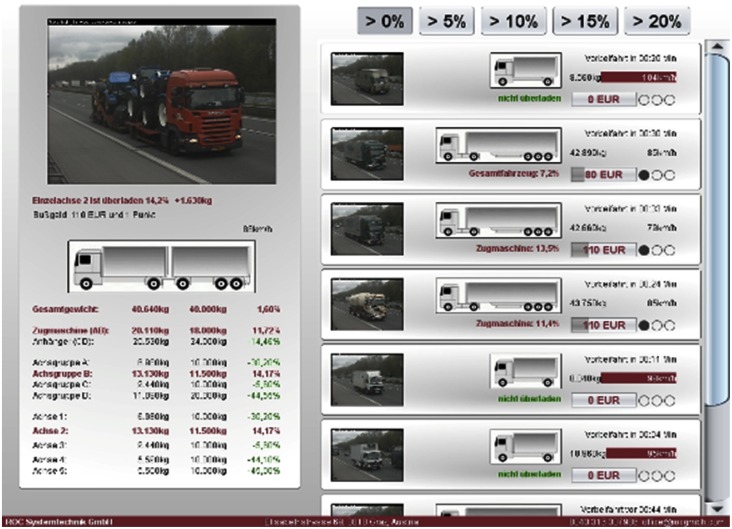
Weigh-in-motion measurement application—station near Rosenheim (Germany).

**Figure 15.**
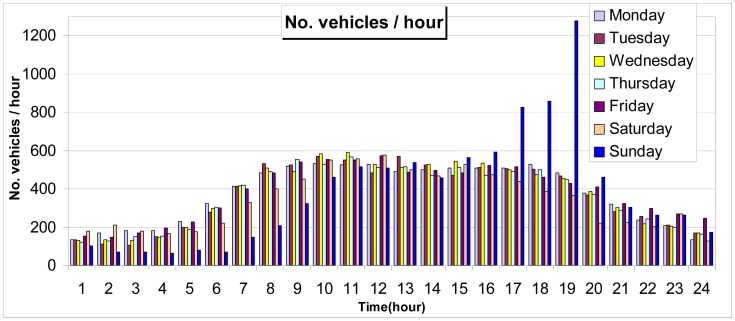
Number of vehicles/hour (hours and days) (1st–7th October 2010).

The weigh-in-motion system mounted in Graz has been working with good results for three years and the one mounted on the highway near Rosenheim for two years, respectively (during this period of time an approximate number of 70,000 vehicles/week have been recorded). Both the sensor and the entire weigh in motion system have proven their reliability during the *in-situ* working period. For the comparison of the new sensor performances with the existing sensors on the market, this one is working in parallel with a bending plate type WIM sensor at the WIM station near Rosenheim. The tests are not yet concluded since the estimated lifetime for the new sensor is of 6–10 years. In Figure 14 the screen of Rosenheim WIM station is presented. High Speed Weigh-in-Motion or HS WIM with its requisite software was developed for semi-automatic overload enforcement (overweight of axles, axle groups, truck, trailer or gross weight) and for application future fully automatic weight control systems. The developed system can detect heavy goods vehicles and overloading in the traffic running on the motorway (see Figure 14). In Figure 15 the number of vehicles/hour recorded by this WIM station, during one week, is presented.

## 9. Conclusions

A new WIM modular and flat shear beam sensor, with a small cross section (90 mm × 24 mm) has been conceived and designed by ROC Systemtechnik GmbH. A Finite Elements Analysis in the elastic range, under static loading conditions, has been performed using the ABAQUS v6.8 software.

For the elastic element of WIM sensor, the design must take into account the maximum von Mises stress, fatigue strength, but also the strains on the strain gage direction and maximum displacement. The most severe state of stress has been obtained for an asymmetric loading of the WIM sensor.

Using the results obtained from the FEA, it has been found out that the sensor could not resist indefinitely (under maximum load of 3,000N on one sensor branch) under fatigue loading. For this reason, the initial design has been substantially modified: the central pin hole has been translated in the area with lower stresses; some connection rays have been increased; for certain surfaces, the machining by grinding has been provided to achieve a better roughness; a steel type with superior characteristics has been adopted; the thickness of external plates has been reduced according to the size of the rolled steel strips existing on the market.

A new FEA has been performed for this improved design, only for the asymmetric loading case. This new alternative of the WIM senor has an infinite fatigue life and a better sensitivity. Accordingly, the WIM sensor has been built up using the new improved design. The weigh-in-motion sensor mounted in Graz has been working with good results for three years and the one mounted on the highway near Rosenheim for two years, respectively. Therefore the authors consider that there are good chances for sensors to have a 6–10 years life span.

## Figures and Tables

**Table 1.** The thickness of the sensor module components and their elastic characteristics.

**No. (see** Figure 3)	**Parts**	**Thickness (mm)**	**Elastic characteristics**	

			**Young modulus E (GPa)**	**Poisson ratio ν**

1 & 5	Outer plates	3.18	200	0.29
2 & 4	Adhesive	0.12	15.2	0.23
3	Central elastic plate	0.5	200	0.29
6	Pin	3 (diameter)	200	0.29

**Table 2.** Main normal stresses and maximum displacements for symmetric and asymmetric loading cases—stainless steel 1.4548.6 (X5CrNiCuNb17-4-4) Bohler N700.

**Loading**	**Thickness [mm]**	**δ_max_ [mm]**	**σ_max_ [MPa]**	**σ_min_ [MPa]**

Symmetric Force (2 × 3,000 N each)	7.1	0.087	395.173	−532.543
Asymmetric Force (1 × 3,000 N)	7.1	0.121	346.833	−711.383

**Table 3.** Maximum stress, minimum stress and maximum displacement of the central plate (asymmetric loading case, Sandvic 7C27M02 steel type).

**WIM shear beam sensor (new design)**	**Total thickness (mm)**	**δ_max_ (mm)**	**σ_max_ (MPa)**	**σ_min_ (MPa)**
	**5.74**	0.131	360.507	−658.179

**Table 4.** Data recorded by the Rosenheim (Germany) WIM station (fragment).

**Point of time**	**hours:min:sec**	**Type**	**Speed (km/h)**	**Axles (No.)**	**Axle weights (kg)**					**Gross weight (kg)**	**Axle distances (mm)**				**Plausible**	**Relative over-weight**
10/1/2010 0:00	0:01:08	120	95	2	6,700	11,420				18,120	5,910				1	0.0066
10/1/2010 0:00	0:01:18	1	88	2	450	450				900	2,500				1	−0.7428
10/1/2010 0:00	0:01:51	98	85	5	6,720	10,140	6,000	5,780	5,690	34,330	3,710	5,570	1,280	1,140	1	−0.0633
10/1/2010 0:00	0:01:57	1	104	2	450	450				900	2,500				1	−0.7428
10/1/2010 0:00	0:02:13	98	88	5	5,940	3,990	1,720	1,900	1,920	15,470	3,550	3,990	1,330	1,330	1	−0.406
10/1/2010 0:00	0:02:16	97	88	4	4,480	3,930	2,310	2,280		13,000	2,960	5,920	1,180		1	−0.5327
10/1/2010 0:00	0:02:19	98	88	5	7,550	9,820	6,660	5,520	6,920	35,840	3,700	5,330	1,330	1,330	1	−0.035
